# Post-Diapause DNA Replication during Oogenesis in a Capital-Breeding Copepod

**DOI:** 10.1093/iob/obad020

**Published:** 2023-06-12

**Authors:** K J Monell, V Roncalli, R R Hopcroft, D K Hartline, P H Lenz

**Affiliations:** Department of Ecology and Evolutionary Biology, University of Michigan, Ann Arbor, Michigan 48109, USA; Pacific Biosciences Research Center, University of Hawai'i at Mānoa, Honolulu 96822, USA; Stazione Zoologica Anton Dohrn, Integrative Marine Ecology, Campania 80121, Napoli, Italy; Department of Oceanography, University of Alaska, Institute of Marine Science, Fairbanks, Alaska 99775-7220, USA; Pacific Biosciences Research Center, University of Hawai'i at Mānoa, Honolulu 96822, USA; Pacific Biosciences Research Center, University of Hawai'i at Mānoa, Honolulu 96822, USA

## Abstract

In high-latitude environments where seasonal changes include periods of harsh conditions, many arthropods enter diapause, a period of dormancy that is hormonally regulated. Diapause is characterized by very low metabolism, resistance to environmental stress, and developmental arrest. It allows an organism to optimize the timing of reproduction by synchronizing offspring growth and development with periods of high food availability. In species that enter dormancy as pre-adults or adults, termination of diapause is marked by the resumption of physiological processes, an increase in metabolic rates and once transitioned into adulthood for females, the initiation of oogenesis. In many cases, individuals start feeding again and newly acquired resources become available to fuel egg production. However, in the subarctic capital-breeding copepod *Neocalanus flemingeri*, feeding is decoupled from oogenesis. Thus, optimizing reproduction limited by fixed resources such that all eggs are of high quality and fully-provisioned, requires regulation of the number of oocytes. However, it is unknown if and how this copepod limits oocyte formation. In this study, the phase in oocyte production by post-diapause females that involved DNA replication in the ovary and oviducts was examined using incubation in 5-Ethynyl-2′-deoxyuridine (EdU). Both oogonia and oocytes incorporated EdU, with the number of EdU-labeled cells peaking at 72 hours following diapause termination. Cell labeling with EdU remained high for two weeks, decreasing thereafter with no labeling detected by four weeks post diapause, and three to four weeks before spawning of the first clutch of eggs. The results suggest that oogenesis is sequential in *N. flemingeri* with formation of new oocytes starting within 24 hours of diapause termination and limited to the first few weeks. Lipid consumption during diapause was minimal and relatively modest initially. This early phase in the reproductive program precedes mid-oogenesis and vitellogenesis 2, when oocytes increase in size and accumulate yolk and lipid reserves. By limiting DNA replication to the initial phase, the females effectively separate oocyte production from oocyte provisioning. A sequential oogenesis is unlike the income-breeder strategy of most copepods in which oocytes at all stages of maturation are found concurrently in the reproductive structures.

## Abstract (it)

Negli ambienti ad alta latitudine, dove i cambiamenti stagionali includono periodi di condizioni difficili, molti artropodi entrano in diapausa, un periodo di dormienza regolato da ormoni. La diapausa è caratterizzata da un abbassamento del metabolismo, un aumento della resistenza allo stress ambientale e da un arresto dello sviluppo. La diapausa permette ad un organismo di ottimizzare i tempi della riproduzione sincronizzando la crescita e lo sviluppo della prole con periodi di elevata disponibilità di cibo. Nelle specie che entrano in dormienza come preadulti o adulti, la fine della diapausa è caratterizzata dalla ripresa dei processi fisiologici, da un aumento dei tassi metabolici e, una volta passata all“età adulta per le femmine, dall”inizio dell“oogenesi. In molti casi, gli individui ricominciano a nutrirsi e le risorse appena acquisite diventano disponibili per supportare la produzione di uova. Nell”oceano subartico, l“alimentazione del copepode *Neocalanus flemingeri* è dissociata dall”oogenesi e la riproduzione è limitata dalle risorse fisse ottenute durante la pre-diapausa. Tuttavia, non è noto come questo copepode regoli la formazione di ovociti per garantire che tutte le uova siano di alta qualità e ben fornite. In questo studio, la fase nella produzione di ovociti da parte di femmine post-diapausa che coinvolge la replicazione del DNA nell“ovaio e negli ovidotti, è stata esaminata utilizzando l”incubazione in 5-Ethynyl-2′-deoxyuridine (EdU). Sia l“oogonia che gli ovociti incorporavano EdU, con il numero di cellule colorate con EdU che raggiungeva il picco a 72 ore dopo la fine della diapausa. La colorazione delle cellule con EdU è rimasta elevata per due settimane, diminuendo successivamente senza alcuna colorazione rilevata entro quattro settimane dopo la diapausa e da tre a quattro settimane prima della deposizione del primo gruppo di uova. I risultati suggeriscono che l”oogenesi è sequenziale in *N. flemingeri* con la formazione di nuovi ovociti che iniziano entro 24 ore dalla fine della diapausa e sono limitati alle prime settimane. Il consumo di lipidi durante la diapausa era inizialmente minimo e relativamente modesto. Questa prima fase del programma riproduttivo precede la metà dell“oogenesi e la vitellogenesi 2, quando gli ovociti aumentano di dimensioni e accumulano le riserve vitelline e lipidi. Limitando la replicazione del DNA alla fase iniziale, le femmine separano efficacemente la produzione di ovociti dall”approvvigionamento di ovociti. Un'oogenesi sequenziale è diversa dalla strategia di riproduzione della maggior parte dei copepodi che mantengono contemporaneamente gli ovociti in tutte le fasi di maturazione nelle strutture riproduttive.

## Abstract (pt)

Em ambientes de alta latitude, onde as mudanças sazonais incluem períodos de condições adversas, muitos artrópodes entram em diapausa, um período de dormência regulado por hormônios. A diapausa é caracterizada por metabolismo muito baixo, resistência ao estresse ambiental e interrupção do desenvolvimento. Ele permite que um organismo otimize a reprodução sincronizando o crescimento e desenvolvimento da prole com períodos de alta disponibilidade de alimentos. Em espécies que entram em dormência como pré-adultos ou adultos, o término da diapausa é marcado pela retomada dos processos fisiológicos, um aumento nas taxas metabólicas e o início da oogênese. Em muitos casos, os indivíduos começam a se alimentar novamente e os recursos recém-adquiridos ficam disponíveis para abastecer a produção de ovos. No oceano subártico, a alimentação do copépode *Neocalanus flemingeri* é dissociada da oogênese e a reprodução é limitada por recursos fixos obtidos durante a pré-diapausa. No entanto, não se sabe como este copépode regula a formação de ovócitos para garantir que todos os óvulos sejam de alta qualidade e bem fornecidos. Neste estudo, a fase de produção de oócitos por fêmeas pós-diapausa foi examinada usando incubação em 5-Etinil-2′-desoxiuridina (EdU) para caraterizar a replicação do DNA no ovário e nos ovidutos. Tanto as oogônias quanto os oócitos incorporaram EdU, com o número de células marcadas atingindo o pico 72 horas após o término da diapausa. A marcação das células com EdU permaneceu alta por duas semanas, diminuindo na terceira e cessando na quarta semana. Desova da primeira ninhada de ovos ocorre três a quatro semanas depois. Os resultados sugerem que a oogênese é sequencial em *N. flemingeri* com a formação de novos oócitos começando dentro de 24 horas após o término da diapausa e limitado às primeiras semanas. O consumo de lipídios durante a diapausa foi mínima e relativamente modesto inicialmente. Essa fase inicial do programa reprodutivo precede a vitelogênese 2, quando os ovócitos aumentam de tamanho e acumulam reservas de vitelo e lipídios. Ao limitar a replicação do DNA à fase inicial, as fêmeas efetivamente separam a produção de oócitos do seu fornecimento. Uma oogênese sequencial é diferente da estratégia de reprodução da maioria dos copépodes que mantem oócitos em todos estágios de maturação nas estruturas reprodutivas.

## Abstract (de)

In Umgebungen in denen der jahreszeitliche Zyklus extreme Bedingungen einschließen, treten viele Arthropoden in die Diapause ein, eine Ruhephase, die hormonell reguliert wird. Die Diapause ist durch einen sehr niedrigen Metabolismus, Widerstandsfähigkeit gegenüber Umweltstress und Entwicklungsstillstand gekennzeichnet. Es ermöglicht einem Organismus, den Zeitpunkt der Fortpflanzung zu optimieren, indem das Wachstum und die Entwicklung der Nachkommen mit Zeiten hoher Nahrungsverfügbarkeit synchronisiert werden. Die Wiederaufnahme physiologischer Prozesse, einen Anstieg des Metabolismus und der Beginn der Oogenese charakterisiert die Beendigung der Diapause bei Arten, die im Vor- oder Erwachsenenalter in den Ruhezustand eintreten. Die Eierproduktion bei vielen Tieren erfordert neue Ressourcen, allerdings gilt dies nicht für alle. Nahrungsaufnahme des Copepoden *Neocalanus flemingeri* ist von der Oogenese entkoppelt, und Reproduktion ist begrenzt durch feste Ressourcen, die während der Prä-Diapause gewonnen wurden. Es ist jedoch unbekannt, ob und wie dieser Ruderfußkrebs die Eizellenbildung einschränkt damit all Eizellen von hoher Qualität und vollständig versorgt sind. In dieser Studie wurde die Phase der Eizellenproduktion bei Weibchen nach der Diapause, die DNA-Replikation im Eierstock und in den Eileitern beinhaltete, durch Inkubation in 5-Ethinyl-2′-desoxyuridin (EdU) untersucht. Sowohl Oogonien als auch Eizellen bauten EdU ein, wobei die Anzahl der EdU-markierten Zellen 72 Stunden nach Beendigung der Diapause ihren Höhepunkt erreichte. Die Markierung der Zellen mit EdU blieb zwei Wochen lang hoch und nahm danach ab, und beendete vier Wochen nach der Diapause. Laichen des ersten Geleges folgt drei bis vier Wochen später. Die Ergebnisse deuten darauf hin, dass die Oogenese bei *N. flemingeri* sequenziell verläuft und die Bildung neuer Eizellen innerhalb von 24 Stunden nach Beendigung der Diapause einsetzt und auf die ersten paar Wochen beschränkt ist. Der Lipidverbrauch während der Diapause war minimal und anfangs relativ bescheiden. Diese frühe Phase des Fortpflanzungsprogramms geht der mittleren Oogenese und Vitellogenese 2 voraus, wenn die Eizellen an Größe zunehmen und Dotter- und Lipidreserven ansammeln. Durch die Beschränkung der DNA-Replikation auf die Anfangsphase trennen die Weibchen effektiv die Eizellenproduktion von der Eizellenversorgung. Eine sequentielle Oogenese unterscheidet sich von der Einkommensbrüterstrategie der meisten Copepoden, bei denen Eizellen in allen Reifungsstadien gleichzeitig in den Fortpflanzungsstrukturen gefunden werden.

## Introduction

The arthropods are a diverse and successful group of metazoans that are an excellent model for the study of the evolution of life-history strategies across habitats (Stearns 2000; Llodra 2002; Chapin 2017). One active area of research is the timing and energetics of reproduction in cyclical environments (Ejsmond et al. 2015; Varpe and Ejsmond 2018; Watrous et al. 2021). Arthropods that inhabit high-latitudes time reproduction and growth to coincide with brief periods of seasonally-driven peaks in productivity. In these environments, arthropods undergo a dormancy called diapause, which is a hormonally-controlled alternate developmental program (Denlinger et al. 2012). The diapausing phase can occur at many different stages during development (embryos, pupae, juveniles, or adults) depending on taxon. In species that diapause as juveniles and adults, individuals build lipid stores prior to entering the dormant phase (Miller et al. 2000; Sim and Denlinger 2013; Denlinger and Armbruster 2014). During diapause individuals are characterized by very low metabolic rates, arrested development, and an increase in lifespan (Hahn and Denlinger, 2011; Denlinger and Armbruster, 2014). Emergence from diapause involves restarting development, resumption of feeding (in some), and activation of the reproductive program (Tauber and Tauber 1976; Tauber and Tauber 1981; Hirche 1996; Denlinger and Armbruster 2014; Lenz and Roncalli 2019).

Arthropods vary in how they meet the cost of reproduction. Breeding strategies range from income to hybrid to capital breeding (Bonnet et al. 1998; Sainmont et al. 2014; Ejsmond et al. 2015; Carver et al. 2021). Diapausing arthropods like mosquitoes are income breeders and fuel reproduction by feeding post-diapause (Denlinger and Armbruster 2014). However, in some arthropods feeding and reproduction are decoupled (Hall 2007; Hirche 2013; Lenz and Roncalli 2019). This “capital-breeding” strategy is employed by some moths (e.g., saturniids, such as the luna moth, [*Actias luna*: Pan and Telfer, 1996; Tuskes et al 1996; Hall 2007; Telfer 2009]). Adults of saturniids lack or have reduced mouth parts with no digestive system, which effectively separates resource acquisition from resource use (Pan and Telfer 1996; Tuskes et al 1996; Telfer 2009). The “capital-breeding” strategy is not limited to moths; capital breeders among the calanoid copepods include species in the genus *Neocalanus* with reduced mouthparts, and species like *Calanus hyperboreus* that resume feeding only after reproduction (Hirche 2013; Lenz and Roncalli 2019). The dependence of post-diapause reproduction on pre-diapause resources is poorly understood. Such an analysis would benefit from a model system in which breeding strategies range from income to capital breeding in closely-related taxa that occupy similar niches. Such a model system exists among small planktonic crustaceans in the family Calanidae (Copepoda: Calanoida) (Table 1).

**Table 1:** Life histories and breeding strategy in the Calanidae.

Breeding	Lifespan (years)	Species	Program	Diapause stage	Reference
Income	<1	*Calanus helgolandicus*	Direct		Bonnet et al. 2005
Income	<1	*Calanus pacificus*	Direct		Baumgartner and Tarrant 2017
Income	<1	*Calanus marshallae*	Direct		Peterson 1988
Income	<1	*Neocalanus gracilis*	Direct	None	Shimode et al. 2009
Hybrid?	≤1	*C. helgolandicus*	Diapause	C5	Bonnet et al. 2005
Hybrid?	≤1	*C. pacificus*	Diapause	C5	Baumgartner and Tarrant 2017
Hybrid	≤1	*C. finmarchicus*	Diapause	C5	Hirche 1997, Marshall and Orr, 1952, and Niehoff et al. 2002
Hybrid	≤1	*C. marshallae*	Diapause	C5	Peterson 1988
Capital	1	*Neocalanus plumchrus*	Diapause	C5	Kobari and Ikeda 2001a; Tsuda et al. 2015
Capital	1–2	*Neocalanus cristatus*	Diapause	C3, C4, C5	Kobari and Ikeda 1999; Tsuda et al. 2015
Capital	1–2	*Neocalanus flemingeri*	Diapause	C4, C6 female	Kobari and Ikeda 2001b; Tsuda et al. 2001; 2015
Hybrid	1–3	*Calanus glacialis*	Diapause	C4–C6	Kosobokova 1999
Capital	>2	*Calanus hyperboreus*	Diapause	C3–C6	Hirche 2013

Copepod species in the family Calanidae include those that are income, hybrid, and capital breeders (Table 1). In general, diapause is facultative in calanids, and many species have both direct-developing and diapausing generations. However, in the subarctic and Arctic, direct-developing individuals have not been reported in species with one-year or longer life cycles in their natural habitat. Direct-developing individuals are income breeders, while individuals that undergo diapause depend on stored energy to fuel reproduction in part or in some cases entirely. In species like *Calanus finmarchicus*, the creation of oogonia and early oocyte development as defined and reviewed by Niehoff (2007) can proceed in the absence of food, but new resources are required to complete oogenesis (Niehoff 2000). In contrast, *Calanus glacialis* can complete its initial bout of oogenesis using only stored lipids, thus allowing this species to spawn before the spring phytoplankton bloom (Niehoff et al. 2002). However, to maximize fecundity upon emergence from diapause *C. glacialis* requires new resources (Hirche and Kattner 1993). In addition, several calanid species are capital breeders and rely on lipid stores accumulated during the prior year to fuel both diapause and reproduction (Table 1). The three *Neocalanus* species in this category have non-feeding adults, and reproduction coincides with end-of-life (Miller and Clemons 1988; Miller and Nielsen 1988; Tsuda et al. 1999, 2001). *Calanus hyperboreus* females represent a different type of capital breeding strategy. They reproduce during the winter using stored lipids, then resume feeding during the spring and summer and reproduce again during the following winter (Hirche 2013). Little is known about how these capital breeders regulate fecundity to maximize the number of fully-provisioned eggs.

In calanoid copepods the creation and maturation of oocytes is continuous, similar to a conveyor belt with most or all stages of oocyte maturation present in the ovary and oviducts concurrently (Hilton 1931; Blades‐Eckelbarger and Youngbluth 1984; Eckelbarger and Blades-Eckelbarger 2005; Niehoff 2007). Early oocytes start at the posterior end of the ovary and move anteriorly through the oviducts as oocytes mature. Oocyte production starts in the germinative zone or the multiplication zone, which is located in the extreme posterior end of the ovary where oocytes begin as oogonia that are mitotically dividing (Hilton 1931). The germinative zone can be identified by the presence of small cells in high densities occupying a rounded outgrowth at the posterior end of the ovary (Eckelbarger and Blades-Eckelbarger 2005). Also situated within the germinative zone are the primordial germ cells, described as one or two larger cells (Hilton 1931). Oogonia may undergo multiple mitotic divisions, and after their final mitosis they become oocytes (Hilton 1931). Oocytes initiate meiosis 1 by undergoing a final bout of DNA replication (“S-phase”) and stopping in prophase 1. These oocytes are found slightly anterior to the oogonia, within an area of the ovary called the synapsis zone (Hilton 1931; Blades‐Eckelbarger and Youngbluth 1984). DNA replication during mitosis (oogonia) and meiosis (oocytes) is expected to occur in the ovary within the multiplication zone and the synapsis zone, respectively. Oocytes continue to move through the oviducts and the anterior regions of the ovary, growing in size through the maturation stages as they accumulate yolk (Blades-Eckelbarger and Youngbluth 1984). Using traditional light microscopy, oocytes can be identified by their color, clear, or tan prior to yolk formation and a golden brown later in development (Blades-Eckelbarger and Youngbluth 1984).

**Fig. 1:**
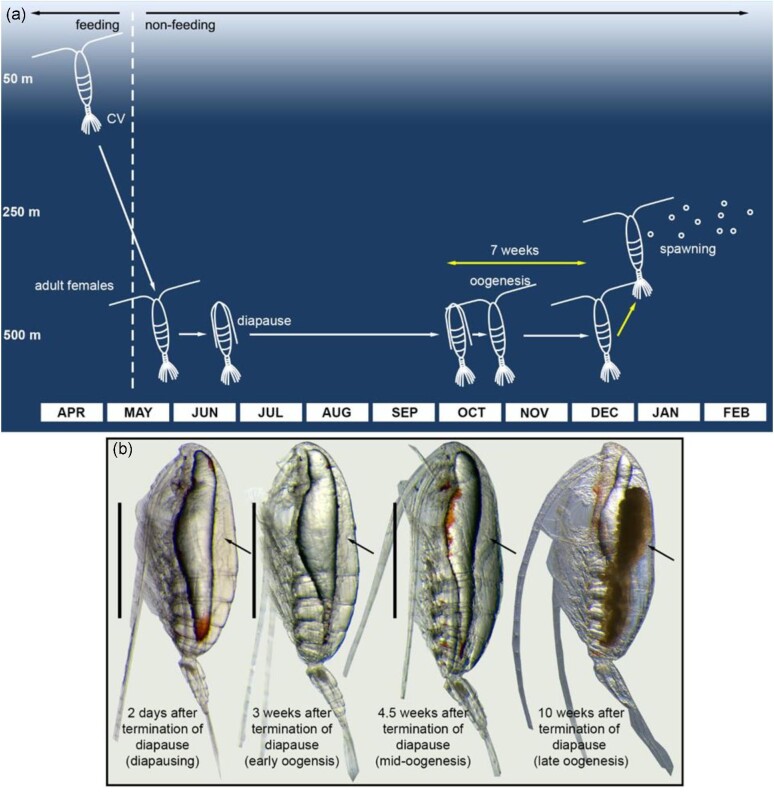
**Diagram of *Neocalanus flemingeri* life cycle in the Gulf of Alaska with images showing morphological changes as oogenesis progresses. (a)** Diagrammatic representation of the seasonal progression of life stages and vertical location of *N. flemingeri* between late April and the following February. The *x*-axis shows time in months, and depth is indicated along the *y*-axis (depths are not to scale). Presence and location of late copepodites (stage CV), adults females (non-feeding), and eggs (embryos) are shown. Yellow arrows indicate known timing of events; white arrows provide estimates for life-cycle transitions. Black arrows mark when feeding individuals transition to non-feeding. The time delay between initiation of oogenesis and spawning is indicated with the double-headed yellow arrow. Life cycle and timing of transitions based on descriptions in Miller and Clemons (1988), Coyle and Pinchuk (2003), and Liu and Hopcroft (2006). Progression from diapause termination to spawning based on Roncalli et al. (2018). **(b)** Light microscope images of *N. flemingeri* females highlighting morphological changes to the ovary as oogenesis progresses. Arrows point to the ovary. Scale bars are 1000 µm.

During periods of starvation, egg production ceases in income breeders like *C. finmarchicus*, but resumes within days after feeding is re-initiated (Hirche 1989; Niehoff 2000). This contrasts with the seven to eight-week delay between emergence from diapause and spawning in *N. flemingeri* at similar temperatures (5–6°C) (Fig. 1—Life history of *N. flemingeri*). In *C. finmarchicus*, oogonia and early oocytes continue to be present throughout starvation, but more mature oocytes undergo atresia, which is a process whereby oocytes disintegrate or disappear from the diverticula (Niehoff 2000). The strategy of resorption of developing oocytes, while maintaining a reservoir of oocytes in the ovary allows the copepods to rapidly resume egg production after food is restored, reaching maximum egg production within weeks. However, resorption of oocytes is energetically expensive, and in a capital breeder could lower lifetime fecundity (Rosenheim et al. 2000). Nevertheless, atresia is common in repeat spawners like herring (Miranda et al. 1999; Kurita et al. 2003; Kennedy et al. 2009). However, for organisms where reproduction and end-of-life coincide, reabsorbed nutrients from atresia would not be available for future reproduction. A better strategy would be to regulate fecundity at the earliest stages of oogenesis, i.e., limiting the number of oogonia, mitotically active germ cells, early in the process in order to maximize the production of fully-provisioned eggs.

Our study focused on *N. flemingeri*. This copepod is ecologically important and a spring biomass dominant in the Gulf of Alaska (Coyle and Pinchuk 2003, 2005; Coyle et al. 2013). It has a one-year lifespan with the primary period of growth and development occurring in the early spring (Miller and Clemons 1988). By late May/June, *N. flemingeri* disappear from the upper 100 m and non-feeding adults accumulate at depth (Fig. 1) (Cooney et al. 2001). Males die after mating, while females migrate deeper and enter diapause at depths below 300 m for *ca.* 5 months (Fig. 1). Emergence from diapause is characterized by the activation of the reproductive program seven to eight weeks before spawning multiple batches of eggs between January and March (Miller and Clemons 1988; Saito and Tsuda 2000; Roncalli et al. 2018, 2020).

Fecundity may be regulated at the oogonia stage in this subarctic species, based on the observation that genes associated with germline formation were down-regulated weeks before the beginning of spawning (Roncalli et al. 2018, 2020). In this study, we tested the hypothesis that oocyte formation proceeds by a discrete sequence of stages after emergence from diapause in this capital-breeding copepod, rather than all stages co-occurring as a continuous ongoing process. This was done by examining DNA replication in the ovary as a proxy for new oocyte formation in *N. flemingeri* females. Newly formed oocytes were tracked by quantifying cells undergoing DNA replication in the ovary of post-diapause individuals collected from depth and incubated in the laboratory for over a month. Concurrent measurements of lipid sac contents were used to estimate resource utilization during the first part of the reproductive program.

## Materials and methods

### Sample collection and sorting

Copepods were collected in Prince William Sound, Alaska in the summer and fall of 2019 during two of three annual oceanographic cruises of the Northern Gulf of Alaska Long Term Ecological Research (NGA LTER) program (https://nga.lternet.edu/). Females “PWS2/June” were collected on June 30, 2019 at the sampling site PWS2 (Latitude 60° 32.1’N; Longitude 147° 48.2’W) (R/V Sikuliaq, cruise number: SKQ201915S), and the “Pleiades/September” females were collected on September 12 and 13, 2019 at PWS2 and near the Pleiades Islands (Latitude 60° 16.7’N; Longitude 147° 59.2’W) (R/V Tiĝlaxˆ, cruise number: TGX201909). Copepods were collected with a Midi MultiNet (0.25 m^2^ mouth area; 150 µm mesh nets) towed vertically from near the bottom to the surface at 0.5 m/sec. In June, experimental copepods were obtained from the MultiNet side net with a non-filtering cod-end and an integrated tow from near the bottom to the surface. For the September collections, copepods came from the 500–400 m stratum. To confirm all females were in diapause upon collection and transitioning to post-diapause, their posture was checked upon retrieval of the net. Many *N. flemingeri* had their antennules adducted and their urosomes were flexed dorsally, which is the typical diapause posture (Lenz and Roncalli 2019). Concurrent zooplankton collections as part of the NGA LTER program confirmed that no adult female *N. flemingeri* were present in the upper 100 m in either June or September (Hopcroft 2021). The absence of pre-adult individuals in the upper 100 m is consistent with the population being in diapause (Miller and Clemmons 1988).

**Fig. 2:**
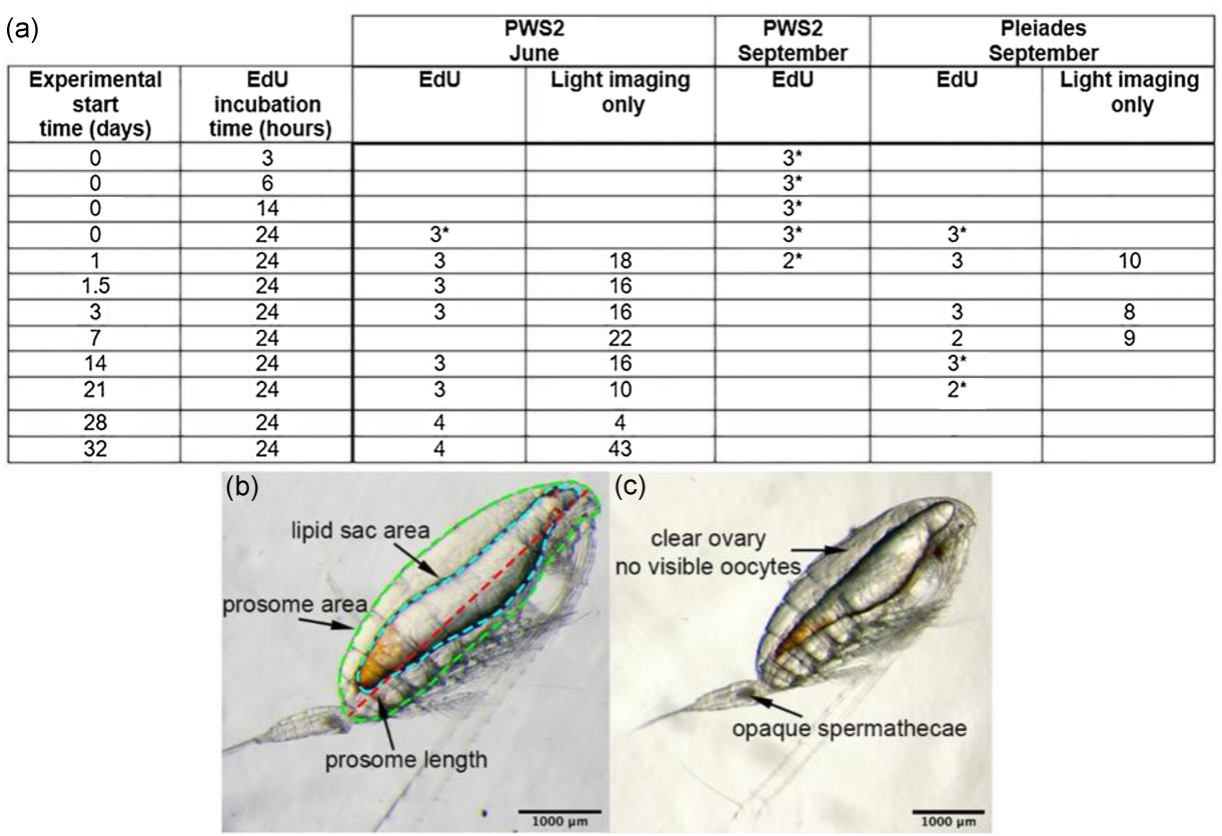
**Summary of *N. flemingeri* experiments completed in the summer (PWS2, June collection) and fall (PWS2 and Pleiades, September collections) of 2019 with light micrographs of females showing image analysis. (a)** Start time and duration of EdU incubations are listed in first two columns. Experimental start time is given relative to collection, starting within two hours after net retrieval. Values indicate number of individuals incubated in EdU and imaged prior to preservation (EdU columns), or number of individuals that were just imaged through a light microscope (light imaging only columns). In a few cases, females incubated in EdU could not be light imaged before preservation and these are indicated by an asterisk (*). **(b**) Female collected from PWS2 in Prince William Sound, Alaska, prosome length (red dashed line), lipid sac area (blue dashed line contour), and prosome area (green dashed line contour) were measured using ImageJ. Lipid sac and prosome area were used to compute total lipid content in mg and % lipid fullness (Vogedes et al., 2010). Pictured female: prosome length = 4.0 mm, prosome area = 4 mm^2^, lipid area = 2 mm^2^, and lipid fullness = 50%. **(c)** Female collected from Pleiades; arrows point to criteria used to select females for experiments. Pictured female: prosome length = 3.8 mm, lipid fullness = 43%. Images were taken three days after collection. Microscope magnification: × 32, scale bars: 1000 µm.

Upon retrieval, net samples were immediately diluted using filtered seawater collected from depth and kept between 4–6°C to minimize thermal stress. All females selected for the experiments were sorted under a dissecting microscope and were checked for the presence of at least one opaque spermatheca as evidence of mating (Fig. 2). A total of 171 females in June and 59 in September were isolated for the experiments. Females were placed in groups of three into 750 mL Falcon tissue-culture flasks and incubated under dim light in an incubator for up to 4.5 weeks following a previously published protocol (Roncalli et al. 2018). Experimental temperatures were at or below deep-water temperatures in Prince William Sound (temperature settings: 4°C for June and 6°C for September). A subset of females was used in the DNA replication experiments; the remaining females were imaged for measurements of prosome length and lipid sac area.

### Experimental design and timeline

The thymidine analogue, 5-Ethynyl-2’-deoxyuridine (EdU) is used to study cell proliferation by measuring DNA synthesis, being incorporated into a cell's DNA during the S phase of the cell cycle. EdU is non-toxic at low concentrations and can be incorporated into an organism while living (Salic and Mitchison, 2008; Beltz et al. 2011; Benton et al. 2014). EdU has been used in other studies that use small aquatic organisms with no deleterious effect when a low dose of EdU is mixed into habitat water (Passamaneck and Martindale, 2012; Zattara and Özpolat, 2021).

For the timeline, two to four females were incubated in low concentrations of EdU, detailed EdU protocol is described below, for 24 hours at eight time points in June (Fig. 2, 0–24, 24–48, 36–60, and 72–96 hours and 2, 3, 4, and 4.5 weeks), and at six time points in September (0–24, 24–48, and 72–96 hours and 1, 2, and 3 weeks) to track the numbers of cells with DNA replication in the ovary and oviducts from collection (diapause) to 4.5 weeks post-collection, corresponding to mid oogenesis based on a previous transcriptomic study (Roncalli et al. 2018). Furthermore, after checking the females from the June experiment, three time points were added in the first 24 hours with shorter EdU incubation periods (0–3, 0–6, 0–14 hours) to establish the start of DNA replication post-diapause. Prior to preservation and processing for confocal microscopy, females were examined by light microscopy for any visible morphological changes and imaged for female size and lipid sac area measurements. Light microscope imaging was not possible for all time points, and those without light imaging are noted in the table in Fig. 2 (asterisks).

### Lipid sac and prosome length imaging

A total of 204 females were imaged for body measurements, with 31 of these females being also part of EdU experiments. Live females were placed in a chilled embryo dish with a small drop of seawater. Females were imaged laterally at 32x magnification using a Leica MZ16 microscope equipped with a 12 MPx Spot Insight camera. Females were checked for signs of damage. The few copepods that had damaged lipid sacs when collected as indicated by the presence of lipid droplets outside the lipid sac were excluded from further analysis. Females with a few broken caudal setae and/or antennules were included but the nature of the damage was noted.

Using ImageJ (Schneider et al. 2012), light microscope images were analyzed manually for three measurements: prosome length in mm, area of the lipid sac in mm^2^, and area of the prosome in mm^2^. Prosome length was measured by placing a line from the anterior to posterior tip of the prosome, measurements were rounded to the nearest 0.1 mm. Lipid sac and prosome area were measured by outlining their perimeters. Total lipid content in milligrams (mg) was estimated using the area of the lipid sac using Vogedes et al. (2010) equation: TL = 0.197A^1.38^, where A is the lipid sac area and TL is total lipid. This relationship was established by comparing lateral images of the lipid sac to gas-chromatographic lipid measurements of three *Calanus* spp. That generated an equation that used lipid sac area as a proxy for lipid content. Measured areas of the lipid sac and prosome were also used to compute a lipid fullness percentage $(\frac{{{\rm{lipid\ sac\ area}}}}{{{\rm{prosome\ area}}}}$ × 100) that incorporates prosome size, since larger copepods can store more lipid than smaller copepods (Miller et al. 2000). Lipid fullness is an index that allows comparisons between individuals while minimizing differences in prosome length, species, and developmental stage (Schmid et al. 2018; Skottene et al. 2019).

### EdU protocol

The EdU incubations at the time points listed above were used to obtain a timeline of the formation of oocytes post-diapause. For each experimental time point two to four females were carefully pipetted out of the experimental flasks, imaged, and transferred into well plates with 2 ml of filtered seawater with 0.5 mg of EdU per copepod in June. This concentration was found to be high, and the EdU concentration was adjusted to decrease labeling brightness. Thus, in September, the concentration of EdU was decreased to 0.06 mg of EdU per copepod. The lower concentration improved viewing in the confocal microscope. Females were incubated in this solution for 24 hours except for the first three September time points (0–3, 0–6, 0–14 hours). After the incubation, females were removed from the EdU, fixed in 4% paraformaldehyde in Sorensen's Phosphate Buffer pH 7.2 (PB) and labeled using a ThermoFisher Click-iT EdU Alexa Fluor 594 Imaging Kit (catalog number: C10639) following the manufacturer's instructions. Samples were washed for 15 minutes thrice in PB then in 0.5% Triton X-100 in PB for three 15-minute long permeabilization washes. EdU-labeled cells were fluorescently tagged with Alexa Fluor 594 dye using a copper-catalyzed click reaction. Three additional 15-minute washes in PB were done before samples were stored in VECTASHIELD Antifade Mounting Medium containing DAPI, a nuclear DNA counter-label to EdU. Samples were stored at 4°C until mounting and imaging. Because DAPI in VECTASHIELD frequently did not permeate into the ovary, dilutions of VECTASHIELD with DAPI or Hoechst 3342 in phosphate-buffered saline were used to fully label the ovary prior to imaging on the confocal microscope.

### Confocal imaging and quantification of cell division

Females were mounted in VECTASHIELD with DAPI with their left lateral side facing up except for three individuals that were mounted dorsally. Samples were imaged using a Leica SP8 X Confocal Laser Scanning microscope with a × 20 glycerol immersion lens and a white light laser. DAPI has an excitation peak of 359 nm and emission peak of 457 nm, while Alexa Fluor 594 has an excitation peak of 590 nm and an emission peak of 617 nm. Imaging was optimized through gating out of some wavelengths to decrease background and autofluorescence. Samples were imaged by tile scanning through each copepod to locate the entire ovary. Z-stack sections were 1.04 µm apart to ensure that no cells were missed due to large imaging gaps. Whole-mount females were imaged from the start of the ovary and oviducts until the depth at which resolution was lost due to insufficient laser penetration. Using Leica's merge software, multiple regions with individual z-stacks were merged to form a single z-stack of an ovary larger than the lens’ field of view.

Cell counts of the ovaries and oviducts were made by projecting each slice of a z-stack onto a monitor and outlining each labeled cell onto tracer paper (Benton et al. 2014). Cells with EdU labeling were then counted through all slices of a z-stack. The structure of the ovary and oviducts could be resolved through the DNA–DAPI label and any cell that was considered in the ovary or oviducts was counted. The ovary and oviducts were identified using previous descriptions of calanoid copepod reproductive structures (Hilton 1931; Blades-Eckelbarger and Youngbluth 1984; Eckelbarger and Blades-Eckelbarger 2005; Niehoff 2007).

## Results

### Female collections and incubations

Adult females collected in June and September showed no signs of oogenesis: their ovaries were clear, and no oocytes were visible during live sorting and initial imaging. While there were no differences in female condition (i.e., signs of stress or damage), differences in long-term survival were observed between the June and September collections. The survival rate for June experimental females was >90% during the 32-day experiment. Collections in September were exposed to high seas throughout the two-week cruise, which most likely contributed to the lower survival (∼60%) of the females.

**Fig. 3:**
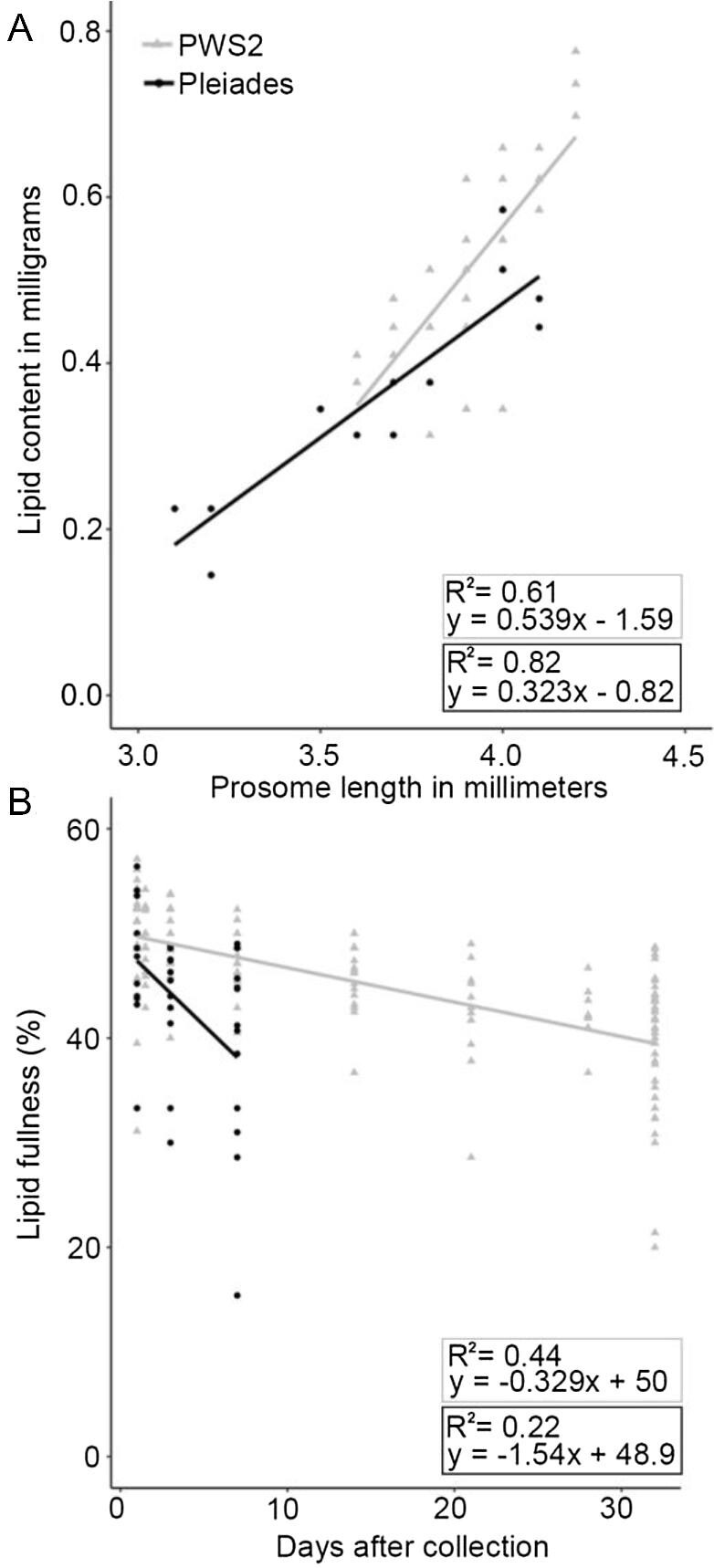
**(A) Scatterplot of prosome length with lipid content from females imaged in the first 36 hours after collection, PWS2/June (grey triangles, n = 40) and Pleiades/September (black circles, n = 13). (B) Scatterplot of female lipid fullness versus days after collection for the duration of the two experiments, PWS2/June (grey triangles, n = 168) and Pleiades/September (black circles, n = 36). (A)** Female prosome length was measured directly from light microscope images and rounded to the nearest 0.1 mm. Lipid content was calculated from measured lipid sac area using Vogedes et al. (2010) equation: TL = 0.197A^1.38^ where A is measured lipid sac area and TL is total lipid content in mg. Separate regressions were computed for the two experimental datasets. **(B)** Lipid fullness is calculated as percentage using the equation, lipid fullness (%) = $(\frac{{{\rm{lipid\ sac\ area}}}}{{{\rm{prosome\ area}}}}$ × 100). Lipid sac and prosome areas were also measured directly from light microscope images. For both collections female lipid fullness decreased with time as shown by the fitted regression lines, which was significant at *p* < 0.001 for PWS2/June and *p* < 0.01 for Pleiades/September.

The range in female sizes collected in June and September was similar, however, on average females from the Pleiades sampling site in September were smaller with mean prosome lengths differing by 0.2 mm between the PWS2/June collection (3.9 mm) and the Pleiades/September (3.7 mm) collection (two sample *t*-test, *p* ≤ .001, *t*[43] = 4.181). On average total lipid was lower in the Pleiades/September females (0.36 mg, s.d. = 0.12 mg, *n* = 13) than in the PWS2/June females (0.53 mg, s.d. = 0.12 mg, *n* = 21; two sample *t*-test, *p* ≤ .001, *t*[26] = 3.894). In contrast, we did not find a significant difference in lipid fullness between PWS2/June (median = 46%, *n* = 21) and Pleiades/September (median = 45%, *n* = 13) females (Wilcoxon Rank Sum Test, W = 3381, *p* = 0.27). In the diapausing females, lipid content and prosome length were not expected to be independent of one another; and these two variables were positively correlated in *N. flemingeri* (Fig. 3a, PWS2/June: *R*^2^ = 0.61; Pleiades/September: *R*^2^ = 0.82). Thus, while females from the PWS2/June and the Pleiades/September collections differed in body size and total lipid content, their lipid fullness was similar, despite the fact that the September females had been in diapause for an additional 2.5 months.

### Lipid utilization during the experimental incubations

Lipid utilization rates were estimated using a regression analysis on relative female lipid fullness over time for each experimental period (Fig. 3b). The regression analysis for the 32-day experimental period of the females collected at PWS2 in June was significant with an estimated lipid loss rate of 2.3% decline in lipid fullness per week (*p* < 0.001). At this loss rate and assuming a linear relationship, lipid reserves in the females would be predicted to be depleted after 152 days for PWS2/June. The regression analysis for the shorter 7-day experimental period of Pleiades/September females was also significant (*p* < 0.01). The estimated loss rate for the females collected in September at the Pleiades station was higher at 10.8% of the lipid fullness per week, a loss rate that would predict depletion of lipid reserves within 32 days.

The PWS2/June and Pleaides/September lipid utilization rates were significantly different from each other (type 2 marginal test, *p* < 0.001).

### Initiation of oogenesis post-collection

**Fig. 4:**
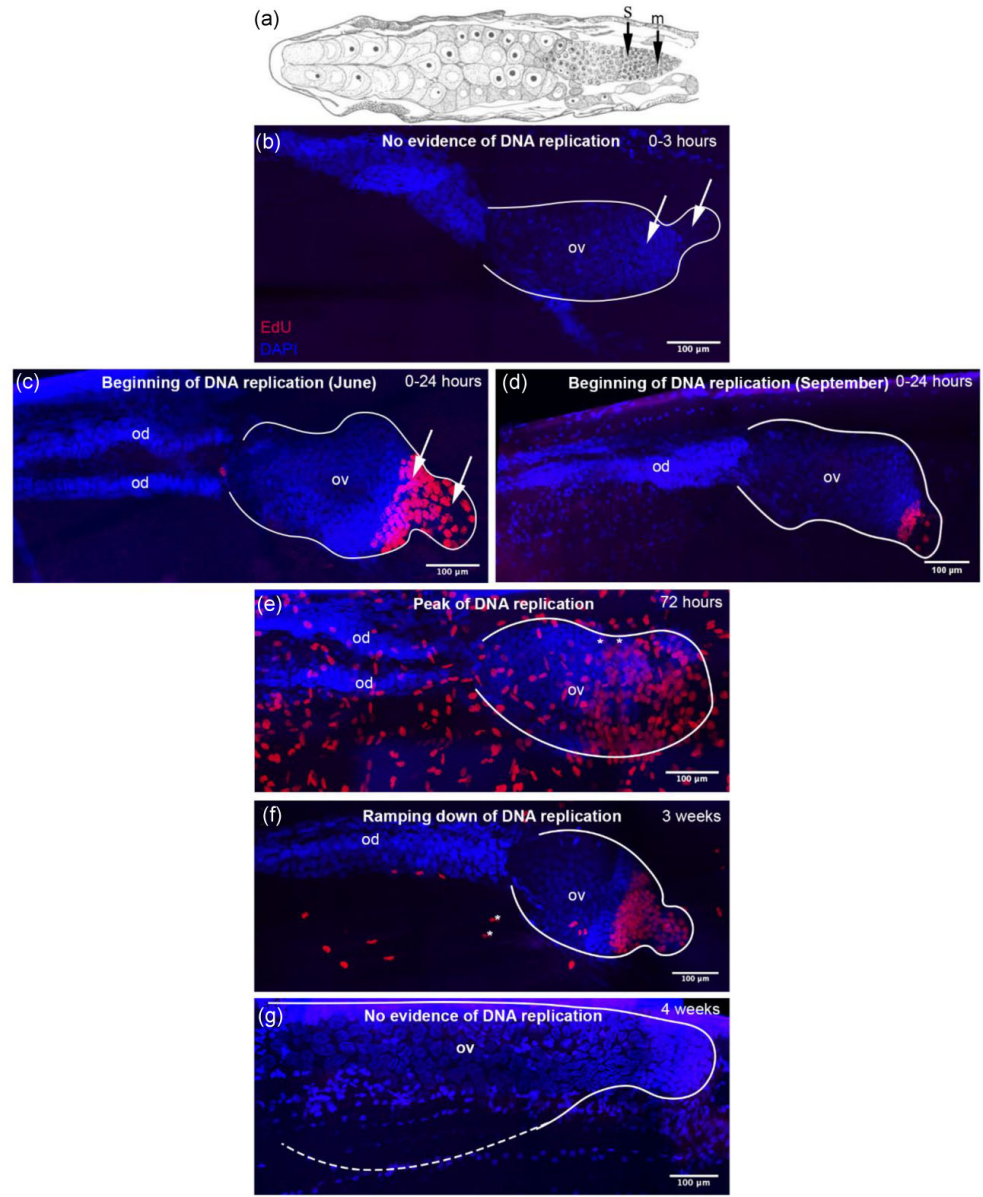
**Maximum Intensity Projections (MIP) of ovaries of females incubated in EdU showing a time series from immediately after collection to four weeks post-collection. (a)** Modified diagram of a section through thorax of *C. finmarchicus* showing ovary and oviducts from Hilton (1931). Arrows point to locations of DNA replication; mitotically dividing oogonia (m: multiplication zone), and oocytes beginning prophase of meiosis 1 (s: synapsis zone). **(b-g)** Images were created using a MIP of merged confocal z-stacks where the brightest voxels over a specified depth are consolidated into one image. All females were collected from the PWS2 sampling site, except for female shown in **d**. Red: EdU-labeled cells, blue: DAPI-labeled cells. In images, cephalosome is to the left; urosome is to the right. White outlines show ovary; ov: ovary, od: oviduct. **(b)** Female was incubated in EdU for three hours directly after collection; Image is a 75 µm projection, number of dividing cells = 0. Arrows point to the approximate locations of the multiplication/germinative zone (oogonia) (right arrows) and the synapsis zone (oocytes) (left arrows). **(c)** Female was incubated in EdU for 24 hours directly after collection in June. Image is a 104 µm projection, number of EdU-labeled cells = 67. **(d)** Female was incubated in EdU for 24 hours directly after collection from in September. Image is a 53 µm projection, number of EdU-labeled cells = 28. **(e)** Female was incubated in EdU for 24 hours at three days after net collection. Image is a 63 µm projection; number of EdU-labeled cells = 295. **: image doubled due to tile merge artifact. **(f)** Female was incubated in EdU for 24 hours at three weeks after net collection. Image is a 36 µm projection; number of EdU-labeled cells = 105. **(g)** Female was incubated in EdU for 24 hours at four weeks after collection. Image is a 10 µm projection; number of EdU-labeled cells = 0. Dotted line indicates shape of ovary not viewable in MIP image. Images were taken at ×20 magnification, scale bars are 100 µm.

Increase in *N. flemingeri* behavioral responsiveness was observed within six hours of collection. All females from both collection sites (*n* = 9) incubated in EdU for 24 hours following collection (0–24 hours) showed evidence for DNA replication in the posterior end of the ovary regardless of sampling site or collection month. To capture the beginning of DNA replication on a finer resolution, we added a set of short incubations in September. None of the females (*n* = 3) incubated in EdU from 0–3 hours post-collection showed any labeling in the ovary (Fig. 4). The first sign of DNA replication was observed in the 0–6 and the 0–14 hour EdU incubations. In each incubation, one of the three females showed presence of EdU labeling within the ovary. Thus, while the first evidence for DNA replication differed among females, all initiated DNA replication between 14 and 24 hours, with a few starting as early as within six hours after collection.

### Location of DNA replication within the ovary

EdU labeling in the ovary and oviducts was predominately localized to the posterior end of the ovary, which is presumably within the germinative zone (Fig. 4). This region was not clearly visible in all females, which was likely a result of the limitations of whole mount confocal microscopy, and not variations in ovary structure. In all except two individuals with a clearly defined germinative zone, EdU incorporation co-occurred both here and in the synapsis zone. EdU incorporation occurred in locations that are consistent with DNA replication occurring in both mitotically-dividing oogonia and oocytes starting meiosis 1. However, we were not able to definitively differentiate between oogonia and oocytes using EdU and DAPI labeling methods. No evidence of large primordial germ cells was seen in this study. We also examined oocytes in the diverticula for any evidence of degradation during the 4.5-week study and found no sign of oocyte resorption.

### Temporal pattern of DNA replication in the ovary

**Fig. 5:**
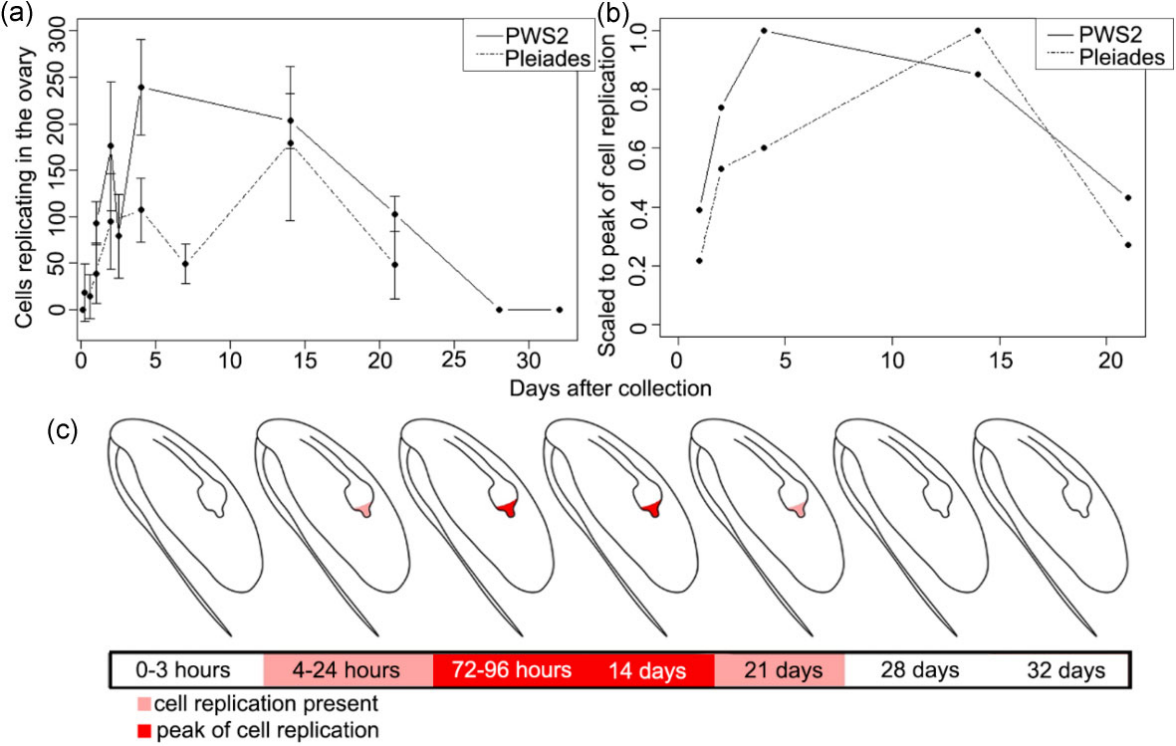
**Description of EdU incorporation into cells within reproductive structures at different times post-collection. (a)** Each data point represents mean number of EdU-labeled cells in the ovary and oviducts averaged across two to six females for each time point. EdU-labeled cells were counted using confocal z-stacks. Error bars are standard deviations. **(b)** Each data point represents normalized EdU-labeled cells computed as the mean number of EdU-labeled cells at a time point divided by that experiment's mean number of cell replications at its peak time point (PWS2/June: 72 hours, 239 cells, dashed line; Pleiades/September: 14 days, 179 cells, solid line). Note difference in x-axis between (a) and (b); (b) only shows time points shared between late June and September, due to this the time points: 1.5, 7, 28, and 32 days are not graphed. **(a, b)** September data (“Pleiades”) also include females collected at the Pleiades, and one set collected at PWS2 (Figure 2). **(c)** Diagram summarizing pattern of EdU incorporation within the ovary of *N. flemingeri* post-collection with transitions noted in time bar along bottom.

The number of cells with DNA replication ramped up quickly after collection and emergence from diapause. Within three days after collection, replicating cells in the ovary had hit a peak in PWS2/June females. While the number of cells with DNA replication varied among females, the peak was sustained for approximately two weeks (Fig. 5). For the Pleiades/September females, a similar initial increase in the number of cells with DNA replication was observed, however, the peak occurred later (two weeks). At three weeks after collection, the number of cells with DNA replication was lower, with a decline of ca. 60% in June and 70% in September. Week three also corresponds to the first time point when females showed morphological changes associated with oogenesis. No DNA replication was observed at 28 and 32 days after collection in any of the PWS2/June females (*n* = 8) regardless of their prosome length (3.5–4.1 mm) or lipid content (0.22–0.44 mg).

### Relationship between the number of cells with DNA replication in ovary and oviducts and female condition

The number of cells with DNA replication was noticeably different between collections even within the first 24 hours. Mean cell counts were consistently lower in Pleiades/September females than PWS2/June ones. Females from both collections were combined to examine if there was a relationship between number of replicating cells in the ovary and oviducts, prosome length, and lipid content in initial conditions. At one day after collection during the ramp up phase, both prosome length (Spearman's rank correlation, $\rho :0.67$, *p* = 0.22, *n* = 5) and lipid fullness (Spearman's rank correlation, $\rho :0.7$, *p* = 0.19, *n* = 5) were positively correlated with the number of cells showing DNA replication. However, this correlation is based on a small number of data points and was not confirmed for later time points.

### No DNA replication observed in oocytes in the oviducts

**Fig. 6:**
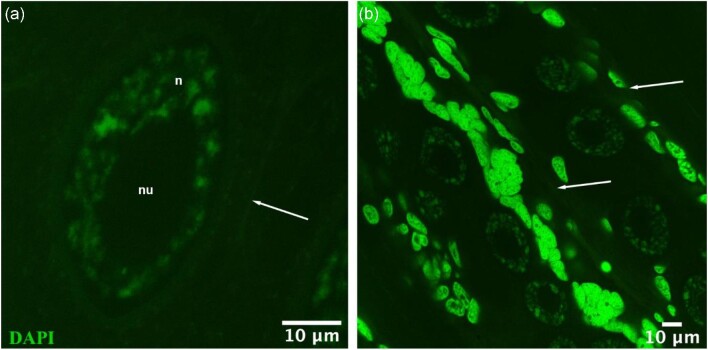
**Confocal images of oocytes with DAPI labeling (green) in oviducts four weeks post collection (PWS2/June).** Green false color used to highlight details. **(a)** Oocytes with condensed chromatin in the nucleus and the presence of a large nucleolus; nu: nucleolus, n: nucleus, arrow: cell boundary. **(b)** Oocytes lined in paired oviducts located between arrows. Arrows point to cellular walls of one of the paired oviducts. Images were taken at × 63 magnification, scale bars are 10 µm.

Older oocytes located in the oviducts or the anterior regions of the ovary were easily identifiable by the presence of a large nucleus, nucleolus, and condensed chromatin (Fig. 6). As expected, EdU labeling was never observed in these older oocytes since they are arrested in meiosis 1. These older DAPI-labeled oocytes were first seen in the oviducts at 21 days after collection. At 28 and 32 days, older oocytes were common in the anterior ovary and oviducts. By this time, oviducts and ovaries had expanded across the prosome to accommodate the large number of growing oocytes.

## Discussion

Oogenesis has been studied using both light and transmission electron microscopy in several copepods, but few studies have targeted capital breeders (Hilton 1931; Blades‐Eckelbarger and Youngbluth 1984; Eckelbarger and Blades-Eckelbarger 2005; Niehoff 2007). This is the first study that examined oogenesis using confocal microscopy and a fluorescent label for DNA replication in *N. flemingeri*. We supported the hypothesis that oogenesis in this capital breeder proceeds through a discrete series of stages, in contrast to being a continuous process as in income-breeding copepods. By using an immunofluorescent labeling technique, we were able to demonstrate that DNA replication within the ovary starts within hours after diapause termination and stops between three and four weeks later, approximately four weeks before females start to spawn. The experimental results and lack of evidence of oocyte resorption suggest that each female creates only as many oocytes as she is able to develop and provision fully. Our results are consistent with field observations of an absence of immature oocytes left in the diverticula (“spent”) of post-reproductive females as they approach end-of-life (Miller and Clemons 1988). The picture that emerges is that this species uses its stored energy efficiently by metering its fecundity during the initial post-diapause phase.

The stress associated with the net collection provides a precise time point for the termination of diapause, which is characterized by an increase in behavioral responsiveness (Lenz and Roncalli 2019; Roncalli et al. 2021). Here, we demonstrated that diapause termination is also associated with the activation of DNA replication and the initiation of oogenesis. We found no evidence of a “refractory period” in either June or September females, unlike what has been reported in insects. During the refractory period, diapausing mosquitos are insensitive to environmental stimuli and will not emerge even after they encounter a diapause termination signal (Denlinger and Armbruster 2014). The absence of a refractory period may be a characteristic of diapause in the family Calanidae. As in our study, calanids like *N. flemingeri, N. plumchrus, C. finmarchicus*, and *C. helgolandicus* all exit the diapause posture and give escape responses within hours after collection (Hirche 1983; Campbell et al. 2004, Lenz and Roncalli 2019). If a refractory period had been present, then we would have expected to capture it, since our two collection times coincided with the beginning and the middle of diapause (Lenz and Roncalli 2019). Unlike insects, diapausing copepods overwinter at depth in an environment that is characterized by constant and stable conditions, and a refractory period may not be necessary.

The EdU-labeling pattern in the ovary indicates that *N. flemingeri* females postpone production of oocytes to post-diapause, and that this process in initiated within 24 hours of diapause termination. However, what is less clear is if DNA replication was restricted to oocytes or if post-diapause oogonia formation occurs. The meticulous light microscopic work by Hilton (1931) established that calanoid ovaries comprise three distinct regions, a posterior multiplication one, a middle narrow synapsis zone, and a broad anterior growth zone leading to the oviducts. The final oogonial mitoses of the multiplication zone occur just posterior to the synapsis zone. The initial Edu-labeling pattern (0–24 hours) included this posterior region. Furthermore, nearly all ovaries imaged with DNA replication had EdU labeling in areas consistent with oogonia division (Hilton 1931) suggesting that both oogonia and oocytes are formed post-diapause in *N. flemingeri*. However, this conclusion is based on location alone, and further studies are needed to determine if and how many times post-diapause oogonia divide before they become oocytes and initiate meiosis.

We explored the possibility of using the EdU labeling data to predict female fecundity but concluded that this is not possible in the current study. Instead, our study identified a number of knowledge gaps on the biology of the copepod. EdU labeling occurs during the DNA synthesis phase (S-phase) in two situations: during mitosis in oogonia and as a step in meiosis-I in oocytes. In oogonia, DNA-replication is soon followed by cell division, whereas in oocytes, the subsequent cell division is delayed until late in development (Blades‐Eckelbarger and Youngbluth 1984; Eckelbarger and Blades-Eckelbarger 2005). Estimates of fecundity require separating DNA-replication in oocytes from oogonia, which we were not able to resolve in this study. Another key parameter for any estimates of fecundity is the duration of the S-phase. In yeast and mammalian cells, the S-phase takes ca. 10 hours (Vanoni et al. 1984, Wartenberg et al. 1998). However, temperature ranges in these studies are much higher (20–38°C), and it is unclear whether the same would hold for *N. flemingeri* females with DNA replication occurring at 5–6°C. Furthermore, the size of the genome can affect the duration of the S-phase, as demonstrated in plants (e.g., Litvinchuk et al. 2007). Copepods in the family Calanidae have large genomes, many exceeding that of humans by a factor of 2 or more (Gregory 2022). While we expect that the 24-hour incubation period used in this study was longer than the duration of the S-phase, this assumption needs to be tested.

A transcriptomic analysis of the *N. flemingeri* reproductive program following diapause proposed that progress through oogenesis was in sequence, unlike other calanoids (Niehoff 2007; Roncalli et al. 2018). In direct-developing calanoid copepods oocyte production is initiated in the pre-adult stage (OS1) and oocytes at all stages of development are typically found in adult females (Niehoff 2007). While spawning ceases during starvation, given food it resumes within days and returns to a plateau within one to two weeks (Niehoff 2000). The rapid return to active spawning suggests that ovaries in starving females maintain a reservoir of early oocytes. In contrast, in *N. flemingeri* the reproductive program is much slower and takes seven to eight weeks (Roncalli et al. 2018; Roncalli et al. 2021), and the females postpone the production of oocytes to post-diapause. This may contribute to the long delay between the termination of diapause and spawning. Furthermore, our results are consistent with the up-regulation of genes involved in cell differentiation, cell cycle and germline development that occur during early post-diapause (Roncalli et al. 2021).

There was strong agreement in the timing of events based on our histological observations with that suggested by gene expression studies on this species. Within this study, females were synchronized not just for the initiation of DNA replication but also for its decline by three weeks and cessation by four weeks post-collection in both June and September. The timing of this decline coincides with a sharp down-regulation of Innexin-2, a gene required for early cell germline (Roncalli et al. 2018). Furthermore, the transition between two and four weeks was marked by the presence of larger oocytes appearing in the diverticula of the DAPI-labeled ovaries. The synchronous increase then decrease of new oocytes, the delay in the appearance of large oocytes, and the sequential up- and down-regulation of oogenesis genes is consistent with a reproductive program that progresses in sequence. Oocyte formation occurs during a narrow time window compared with spawning in *N. flemingeri* that can stretch over six weeks and longer (< 28 vs. ∼ 50 days, respectively) (Saito and Tsuda 2000). However, it should be noted that clutch size declines with clutch number, and the two first clutches account for more than 60% of total fecundity (Saito and Tsuda 2000).

Oocyte resorption (atresia) is widespread among both vertebrates and invertebrates (Corriero et al. 2021). It has been reported in capital breeders like herring (Miranda et al. 1999; Kurita et al. 2003; Kennedy et al. 2009) and the income breeding copepod *C. finmarchicus* during starvation (Niehoff 2000). In the latter, oocytes degenerated into vesicles in the diverticula, which was not seen in this study. Toward the end of our experiment, females were transitioning between vitellogenesis 1 and vitellogenesis 2 based on the size of the oocytes, which is consistent with reported expression patterns of genes involved in oocyte differentiation (transcripts encoding Spire, Bicaudal, Cappuccino) and maternal control (transcripts encoding Tudor and Torso) (Roncalli et al. 2018). Atresia is under hormonal control, and it typically includes programmed cell death in mammals and other organisms (Corriero et al. 2021). However, in *N. flemingeri* genes involved in programmed cell death were expressed at low levels until after females started to spawn, when these genes were highly expressed compared with diapausing and pre-spawning females (Roncalli et al. 2020). The exception to this pattern was the up-regulation of apoptosis regulator BAX at week 3 post-collection (Roncalli et al. 2018). This gene belongs to the Bcl-2 protein family, which includes both pro- and anti-apoptotic members (Kornbluth and White 2005). Furthermore, in other eukaryotes, this protein requires post-translational modification to activate its pro-apoptotic function (Borner 2003).

Under resource limited conditions, energy recovered from oocyte resorption can increase longevity and ensure future reproduction. In the case of *C. finmarchicus* energy would be redirected to maintenance and potentially delay egg production until more favorable conditions return (presence of food) (Niehoff 2000). However, oocyte resorption is energetically costly (Rosenheim et al. 2000; Jervis et al. 2008), and reproduction in *N. flemingeri* is linked to end-of-life: an overproduction of early oocytes and oocyte resorption would be energetically inefficient. Lifetime fecundity in this species has been reported as 924 (S.E. = ± 346, Saito and Tsuda 2000) and 535 (S.D. = ± 258, Slater 2004) eggs, which is comparable to other calanoid species (Mauchline, 1998), but is lower than that reported for *C. finmarchicus* in the laboratory under high-food conditions (Niehoff 2000).

Based on lipid fullness, females collected in June at PWS2 and September near the Pleiades were in good to excellent condition, and most females (84%) were considered fully stored (>40% lipid fullness) and none fell into the sparsely-stored category (<4% in lateral view) (Tsuda et al. 2001). Lipid fullness was variable but did not differ between the two sampling times. Our results are in agreement with another report that found that lipid reserves in diapausing *N. flemingeri* were similar between years and also between collection times (July vs. September) (Coleman 2022). These observations suggest that the diapausing females’ use of stored lipids between the end of June and September was very low and consistent with highly reduced metabolic rates. Furthermore, the females may be using other energy sources such as glycogen to fuel diapause before switching to stored lipids (Zhou and Miesfeld, 2009; Roncalli et al. 2020). However, once diapause was terminated, lipid stores declined, which is consistent with the observed up-regulation of genes involved in respiration and lipid catabolism soon after collection (Roncalli et al. 2020, 2021). A question that remains is whether the rate of lipid depletion is constant during post-diapause. Energetic needs might increase during vitellogenesis 2, which occurs at four to five weeks after collection (Roncalli et al. 2018), and is period of rapid yolk accumulation (Blades‐Eckelbarger and Youngbluth 1984; Niehoff 2007).

## Conclusion

The life history of *N. flemingeri* represents one extreme along the income-capital breeding continuum. How this species and other calanids optimize reproduction requires an integrated understanding of resource acquisition and availability, reproductive physiology, population dynamics, and ecology. Oocyte generation and fate within the context of stored and new resources are key aspects that need to be quantified in the natural environment. In this study, *N. flemingeri* females varied in size, lipid reserves, and oocyte formation. While female size and lipid volume were positively correlated, the number of cells showing DNA replication was not consistently related to lipid volume. However, irrespective of lipid content, oogenesis was proceeding in all females as shown by the presence of DNA-replication in cells within the ovary and the presence of maturing oocytes in the diverticula. While the relationship between lipid volume and total fecundity will require further investigation, the variability we observed in the number of cells with DNA-replication is consistent with the range in fecundity reported for this species (Saito and Tsuda 2000; Slater 2004). A comparative analysis across species using EdU would lead to a better understanding of oocyte production, source of energy, and optimization of reproduction. Such an analysis could contribute new insights into the resource utilization and species resilience in a variable and changing environment.

## Data Availability

The data for this article are available at the Biological and Chemical Oceanography Data Management Office, at https://www.bco-dmo.org/project/720280.

## Summary statement

In capital breeders like the planktonic crustacean *Neocalanus flemingeri* resource acquisition is decoupled from egg production. Oocyte production was tracked in post-diapause females using a DNA replication test to better understand how fecundity is optimized.
